# Changes in the Brain Microstructure of Children with Primary Monosymptomatic Nocturnal Enuresis: A Diffusion Tensor Imaging Study

**DOI:** 10.1371/journal.pone.0031023

**Published:** 2012-02-17

**Authors:** Du Lei, Jun Ma, Xiaoming Shen, Xiaoxia Du, Guohua Shen, Wei Liu, Xu Yan, Gengying Li

**Affiliations:** 1 Shanghai Key Laboratory of Magnetic Resonance, Department of Physics, East China Normal University, Shanghai, China; 2 Department of Developmental and Behavioral Pediatrics of Shanghai Children's Medical Center, XinHua Hospital affiliated to Shanghai Jiao Tong University School of Medicine, Shanghai Key Laboratory of Children's Environmental Health, Shanghai, China; Hangzhou Normal University, China

## Abstract

**Background:**

Primary monosymptomatic nocturnal enuresis (PMNE) is a common disorder in school-aged children. Previous studies have suggested that a developmental delay might play a role in the pathology of children with PMNE. However, microstructural abnormalities in the brains of these children have not been thoroughly investigated.

**Methodology/Principal Findings:**

In this work, we evaluated structural changes in the brains of children with PMNE using diffusion tensor imaging (DTI). Two groups consisting of 26 children with PMNE and 26 healthy controls were scanned using magnetic resonance DTI. The diffusion parameters of fractional anisotropy (FA) and mean diffusivity (MD) were subjected to whole-brain, voxel-wise group comparisons using statistical parametric mapping (SPM). When compared to healthy subjects, children with PMNE showed both a decrease in FA and an increase in MD in the thalamus. MD also increased in the frontal lobe, the anterior cingulate cortex and the insula; these areas are all involved in controlling micturition. The significant changes seen in the thalamus could affect both urine storage and arousal from sleep.

**Conclusions/Significance:**

The microstructure abnormalities were observed in the thalamus, the medial frontal gyrus, the anterior cingulate cortex and the insula, which are involved in micturition control network. This indicates developmental delay in these areas may be the cause of PMNE.

## Introduction

Nocturnal enuresis is a common developmental disorder that affects 15–20% of 5-year-old children [1] , and it has important negative effects on the self-image and performance of these children [2]. Enuresis in children without additional lower urinary tract (LUT) symptoms (excluding nocturia) or a history of bladder dysfunction is defined as monosymptomatic nocturnal enuresis [3], while children with both enuresis and any other LUT symptoms are classified as having non-monosymptomatic enuresis [3]. When a child with monosymptomatic enuresis has never had a period of established urinary continence for more than six months, this is considered primary monosymptomatic nocturnal enuresis (PMNE).

Several factors are associated with and contribute to nocturnal enuresis, including heredity, polyuria, detrusor overactivity, sleep and central nervous system mechanisms [4]. In the past, electroencephalograph (EEG) [5], event-related brain potential (ERP) [6], and the startle blink [7] have all been used to study enuresis. It has been observed that the maturational delay of the central nervous system is an important factor in the pathogenesis of nocturnal enuresis [5], [6], [7], because it can induce functional and structural abnormalities in some brain areas. Using functional magnetic resonance imaging (fMRI), a number of studies have reported alterations in several brain functions in patients with urgency and urge incontinence [8], [9]. One study utilized event-related fMRI in PMNE subjects to reveal that children with PMNE had deficits in working memory [10]. We have performed a series of fMRI experiments to investigate the functional abnormalities that are associated with PMNE. In our previous studies, we reported that forebrain activation was altered during a response inhibition task [11] and that spontaneous brain activity changed during the resting state in children with PMNE [12]. These functional changes may originate from a structural abnormality. This idea is supported by a study from Kuchel et al., which has suggested that the presence of white matter hyperintensities seen by magnetic resonance imaging (MRI) both in the right inferior frontal region and in selected white matter tracts predicts the presence and severity of incontinence [13]. We therefore speculate that the structure of the brain is likely abnormal in PMNE patients. However, the exact cerebral areas involved in the pathogenesis of PMNE remain unclear and the brain structure of these patients is poorly understood. Therefore, we evaluated changes the brain structure of children with PMNE using diffusion tensor imaging (DTI).

DTI is an MRI method which provides information about tissue microstructure and its physiologic state [14]. Changes in these parameters can be determined by two important measurements: fractional anisotropy (FA) and mean diffusivity (MD). FA values are most often used to characterize the integrity of white matter tracts, while MD reflects the overall magnitude of diffusional motion within a given voxel. Recently, researchers observed an increase of FA in 11 white matter tracts from childhood to adolescence [15]. Previous research has reported that linear increases in FA and age-related MD decreases are observed in white matter during adolescence [16], [17], [18]. The values of FA and MD would change if the child was developmentally delayed.

In this study, we used statistical parametric mapping (SPM) to perform a whole-brain analysis of the data and to explore structural differences in the brains of children with PMNE and healthy controls. In addition, we also performed a region of interest (ROI)-based analysis of FA and MD to investigate potential changes in the thalamus of children with PMNE.

## Results

When compared to healthy children, children with PMNE demonstrated a significant decrease in FA in the bilateral thalamus, the left frontal lobe (sub-gyral) and the left limbic lobe (uncus).

Children with PMNE also showed an increase in MD in the bilateral anterior cingulate (ACC), the bilateral insula, the right medial frontal gyrus, the right thalamus, the right midbrain, the left anterior lobe of the cerebellum, the right lentiform nucleus and the right sub-lobar (extra-nuclear white matter) when compared to healthy children.

**Figure 1:**
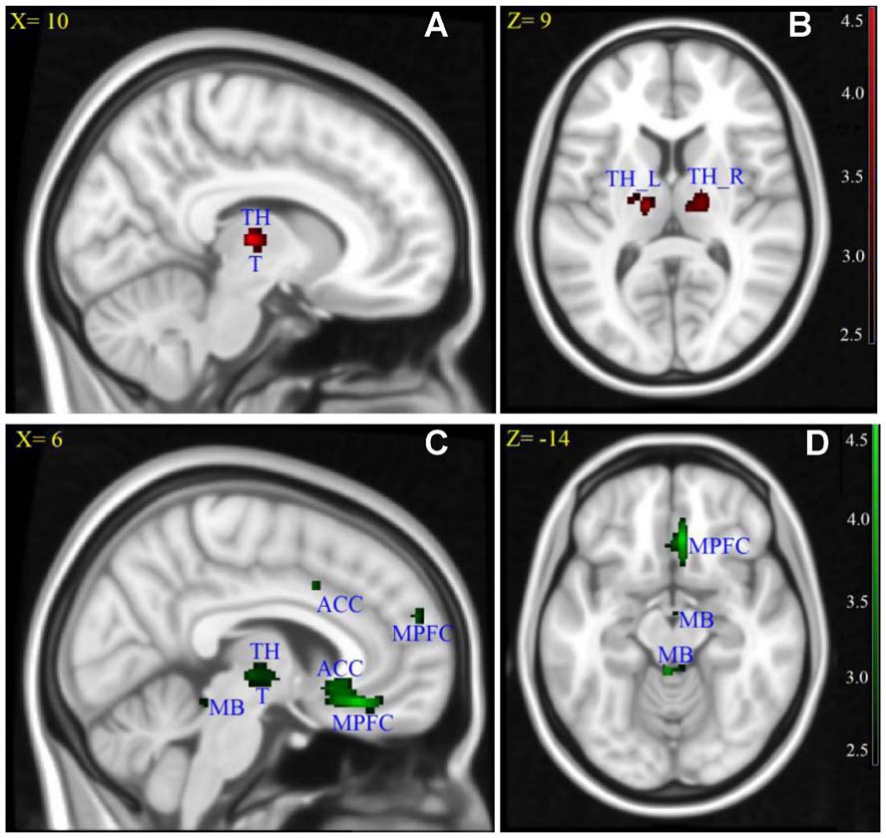
Results of fractional anisotropy (FA) and mean diffusivity (MD) for group analysis between children with PMNE and healthy children. (A) and (B) FA decreases in when compared to healthy children. (C) and (D) MD increases in children with PMNE when compared to TH: thalamus, T: hypothalamus, TH_L: left side of the thalamus, TH_R: right side of the thalamus, MPFC: medial prefrontal cortex, ACC: anterior cingulate cortex, MB: midbrain. Also, X = : X coordinate in MNI space, Z = : Z coordinate in MNI space. (A) and (C) are located in the right brain.

**Figure 2:**
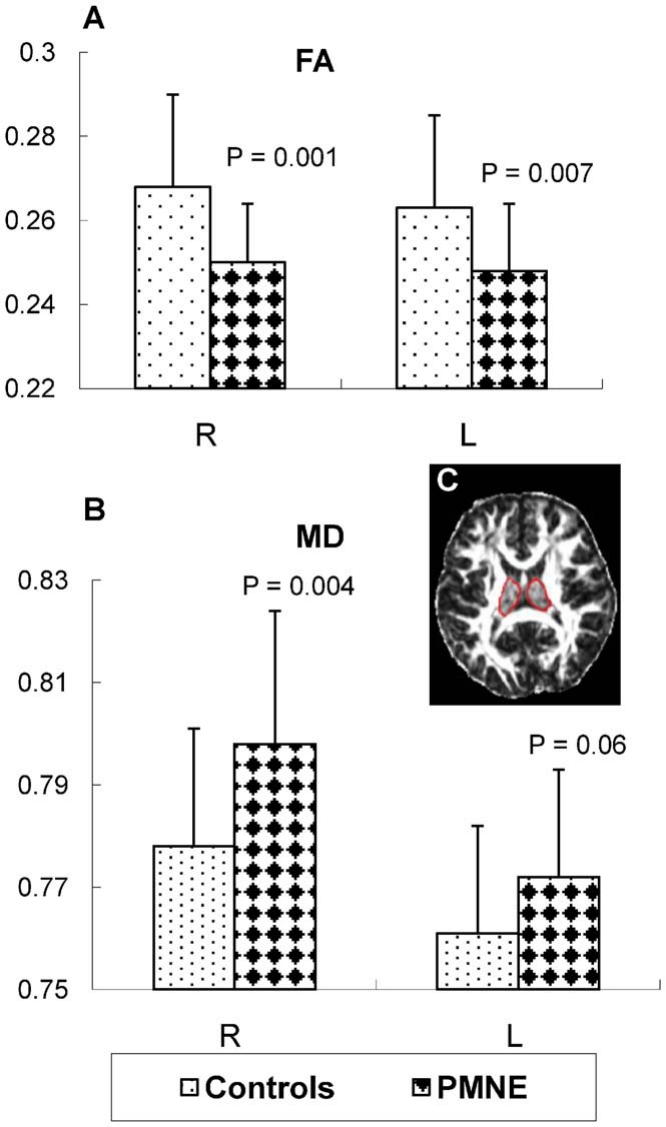
The results of the region of interest (ROI) analysis of fractional anisotropy (FA) and mean diffusivity (MD). P values were analyzed by a two-sample t-test between the two groups. L: left side of the thalamus, R: right side of the thalamus.

A significant decrease in FA and increase in MD were revealed in the thalamus by both an SPM- and an ROI-based analysis. Detailed results are shown in Fig. 1, Fig. 2 and Table 1.

**Table 1:** Results from the SPM analysis of fractional anisotropy (FA) and mean diffusivity (MD).

Predominant regions in the cluster	T value^a^	P value^a^	Number of voxels	Peak location (X Y Z)		
**FA decrease in PMNE**						
Right thalamus	4.33	<0.001	168	10	−18	6
Left thalamus	3.81	<0.001	92	−12	−14	10
Left frontal lobe/sub-gyral	4.04	<0.001	23	−24	12	40
Left limbic lobe/uncus	3.74	<0.001	10	−18	6	−28
**MD increase in PMNE**						
Right medial frontal gyrus	4.50	<0.001	124	4	28	−16
Right anterior cingulate	3.40	<0.001	55	6	18	−8
Right anterior cingulate	3.29	= 0.001	12	8	10	38
Left anterior cingulate	3.63	<0.001	21	−4	26	22
Right thalamus	3.52	<0.001	48	8	−18	0
Right insula	3.49	<0.001	31	34	16	−2
Left insula	3.35	= 0.001	20	−36	20	0
Right medial frontal gyrus	3.33	<0.001	15	8	54	24
Right brainstem/midbrain	3.42	<0.001	29	2	−14	−18
Left cerebellum anterior lobe/midbrain	3.75	<0.001	29	−2	−40	−14
Right lentiform nucleus/lateral globus pallidus	3.52	<0.001	71	16	0	4
Right sub-lobar/extra-nuclear white matter	2.94	<0.001	17	10	6	−4

X, Y, Z = MNI coordinates.

a For peak areas of activation.

## Discussion

When compared to healthy subjects, children with PMNE showed both a decrease in FA and an increase in MD in the thalamus; MD also increased in the medial frontal gyrus, the ACC and the insula, which are all involved in the control of micturition [8]. Previous studies have reported that FA increases and MD decreases with age throughout childhood and adolescence [15], [16], [17], [18]. These changes in FA and MD were observed in children with PMNE but not in healthy children, which suggests a delay in central nervous system maturation in children with PMNE.

### 3.1 Thalamus

All information that reaches the cerebral cortex is relayed by the thalamus including sensation, spatial sense, perceptions and voluntary movements; the thalamus also regulates consciousness, sleep, and alertness [19], [20], [21]. We observed a significant decrease in FA and an increase in MD in the thalamus of children with PMNE, which suggests that the microstructure of the thalamus is abnormal in these patients. The previous research showed that anisotropic increase with age in the anterior thalamic radiations during adolescence, suggesting that the relay between the cortex and thalamus may be undergoing a refinement of connections during adolescence [16].

In the currently accepted model of bladder control, the thalamus plays an important role in relaying the signal coming from the midbrain periaqueductal gray (PAG) by transmitting it to the ACC, the insula and the lateral prefrontal cortex (LPFC) [8], [9]. Previous research has shown that the thalamus is activated during urine storage or bladder infusion [22], [23]. In fact, the thalamus is involved in relaying sensory afferent information from the bladder, which is important during the urine storage phase. The significant reduction in FA and the increase of MD (Fig. 1B) in the thalamus, which may affect thalamic neuronal signal transmission, could influence signal transmission from the PAG to the ACC, the insula and the prefrontal cortex (PFC). If this occurs, urine cannot be properly stored in children with PMNE.

The thalamus also plays an important role in regulating states of sleep and wakefulness and is affected in some sleep disorders [24]. Because children with PMNE do not have involuntary urination during the daytime, a sleep disorder is one of the pathogenic factors of PMNE [25]. Previous research has suggested that an immaturity in the function of the thalamus might be a cause of the arousal dysfunction in patients with Type I enuresis (Type I enuresis was defined as: the cystometrogram is stable and when the bladder is filled, and the EEG pattern changes to that for arousal; and enuresis occurs without the patients awakening.) [26]. A high arousal threshold and an inability to wake during sleep in response to the need to void may directly cause bed-wetting. Therefore, a dysfunctional thalamus may affect the ability of children of PMNE to wake up when they need to void.

A dysfunction in the thalamus could affect both the relay of sensory afferent information that originates in the bladder and the ability to wake during sleep in response to a need to void. Although each of these thalamic dysfunction mechanisms could affect micturition control separately, it is more likely that both of them occur in PMNE. Taken together, these data suggest that a developmental delay or an abnormality of the thalamus could be an important factor behind the bed-wetting seen in children with PMNE.

### 3.2 Medial frontal gyrus

The most significant cluster revealed by SPM was the increase in MD that was observed in the medial frontal gyrus, which suggested a dysfunction of this brain region in children with PMNE. In our previous studies, abnormal activation of the PFC was seen both during a Go/NoGo task and during the resting state in children with PMNE. The PFC has been implicated in planning complex cognitive behaviors, decision making and moderating proper social behavior [27]. A This brain area is connected to the PAG, the ACC, the insula, the hypothalamus and the thalamus; these brain areas are all involved in the control of micturition [8]. Previous brain imaging research has shown that the PFC is activated during both the voiding and filling phases of micturition [8], [9], [28], [29]. In Fowler's preliminary working model of lower urinary tract control by higher brain centers, the medial prefrontal cortex (MPFC) is involved in the storage and voiding phases [8]. Generally, the PFC is associated with decision making in voiding [8], [9]. Patients with bilateral MPFC lesions and multiple sclerosis experience a disturbance in micturition [30], implying that the dysfunction of the MPFC may reduce the ability to withhold urine and cause inappropriate micturition.

### 3.3 Anterior cingulate cortex

The ACC has a wide range of cerebral functions that involve both cognitive function and emotion [31]. This structure may also play a role in mediating bodily arousal states and may take part in interoceptive awareness [32]. Beckel and Holstege have suggested that the ACC is one of most important brain areas that is involved in voluntary voiding and that the ACC may be involved in attention and introspection (i.e., being aware that the bladder is full and that it is time to void) in addition to executive control (i.e., voiding in an “appropriate” place) [33]. Previous research has suggested that ACC responses seem to be associated with pontine micturition center inhibition because reduced ACC activity accompanies the failure of inhibition [34].

In our study, SPM revealed increases in MD in the ACC, suggesting that this abnormality in the ACC might induce micturition at an inappropriate time and place.

### 3.4 Insula

The insula is widely considered to be involved in bladder control. When the bladder is full, the insular cortex is activated in healthy people [22]. Insular and visceral sensations are related; in the insula, the visceral sensation signals are changed into comprehensible consciousness [32], [35]. For example, the insula translates the signal from a fully filled bladder to a conscious internal signal; therefore, the insula aids in interoceptive awareness of bladder states. Our observation that MD increases on both sides of insula may suggest that there are problems in the translation of this signal in the insula. While asleep, the signal from a full bladder might not be transmitted to the brain in time, which would prevent the brain from being aware of the state of the bladder and result in a delay or a missed warning signal indicating that the bladder is full.

### 3.5 Other brain regions

Our results also showed an increase in MD in the right lateral globus pallidus in children with PMNE when compared to healthy children. The globus pallidus has been shown to be activated in normal men during micturition but not at rest [28]. Tai et al. also detected the activation of the globus pallidus during a micturition contraction in rats [36]. Although the activation of this region has been reported in several studies, its exact function in micturition control is still unclear.

Finally, we found two peak areas with increased MD. One was in the midbrain, while the other was near the PAG (x = 2, y = −14, z = −18). It has been established that the PAG plays an important role in bladder control [8]. Moreover, an SPM-based analysis also revealed differences in the uncus, the sub-gyral portion of the left frontal lobe and the midbrain. However, the role of these areas in the pathogenesis of PMNE remains unclear.

Additionally, our results shows difference mainly in gray matter, which is further discussed as following: firstly, DTI measures the restricted diffusion of water through brain tissue, which is sensitive to local microstructure. White matter and gray matter consist of many neuronal processes, which both contribute to water diffusion. It is generally accepted that FA is more sensitive in white matter because of the special structure of nerve fiber. However, FA and MD can also be used to measure microstructure connections of dendrites and soma in gray matter. DTI has been used to detect microstructure changes of gray matter in many researches [37], [38], [39], [40]. Secondly, both gray matter and white matter are involved in structure changes in the maturing brain [40]. However, our results that microstructure changes were primarily in gray matter, may suggest that the gray matter of the PMNE children have more significant abnormality than their white matter. In other words, it suggested that the dysfunction of gray matter, rather than white matter, may play a key role in PMNE. Finally, previous studies showed that thalamus microstructure is involved in developmental changes from children to adults. For example, Snook et al. observed that FA increased and MD decreased in the thalamus of young adults compared to children [40]. Literatures also reported that FA value of thalamus was observed to be abnormal in some disease, such as neurofibromatosis type 1 [41] and autism spectrum disorders [42]. So the significant changes in thalamus that is observed in our experiment suggest that a possible developmental delay or abnormality in the thalamus of PMNE children.

In summary, our results revealed microstructural abnormalities in the thalamus, the medial frontal gyrus, the ACC and the insula of children with PMNE. These areas are all involved in the control of micturition; therefore, a developmental delay in any of these regions may be an important cause of PMNE. In particular, thalamic dysfunction may play an important role in PMNE.

## Materials and Methods

### 4.1 Ethics statement

This study was approved by the Institutional Review Board of Shanghai Children's Medical Center, which is affiliated with Shanghai Jiao Tong University School of Medicine (No: SCMC-201014). All participants involved in our study have known the Informed Consent before the experiments. This consent was written, which was signed by each participant and their guardians with their consent. And the ethics committees approved this consent procedure.

### 4.2 Subjects

We studied 30 children with PMNE and 48 healthy children with the consent of children and their guardians. After exclusion of children with excessive head motion as well as match of age, IQ and sex, 26 children with primary nocturnal enuresis (mean age 10.5; range 7–15; 8 female) and 26 healthy controls (mean age 10.7; range 7–15; 9 female) were available for further analysis. All of the children were right-handed with an IQ>75, and there was no significant difference between the IQs of the two groups (p = 0.218) as assessed by the Wechsler Intelligence Scale for Children revised (WISC-R). The children with PMNE have been excluded suffered from spina bifida occulta by lumbosacral X-ray imaging. And their bladder volumes were measured by b-ultrasound examination. Additionally, all neurological and psychiatric diseases were excluded both by a clinical examination and a structured interview based on the criteria listed in the Diagnostic and Statistical Manual of Mental Disorders (DSM-IV). All of the 26 children with PMNE were outpatients at Shanghai Children's Medical Center. Additional clinical data about the patient group are listed in Table 2.

**Table 2:** Clinical data collected from the patient group.

	Age (years)	Gender	Weight (kg)	Bed-wetting frequency (per day)	Bed-wetting frequency (per week)	Bladder volume^b^(ml)	Frequency of waking up for voluntary voiding
1	10	M	40	1	6	173	Sometimes
2^a^	10	M	30	1	1–2	303	Sometimes
3^a^	10	M	30	1	2	130	Sometimes
4	8	M	35	1	1	100	Often
5	14	M	75	1	2	114	Never
6^a^	11	F	35	1	4	132	Sometimes
7	8	F	25	2	Everyday	140	Sometimes
8	9	M	48	0–1	1	130	Sometimes
9	10	M	29	2	Everyday	100	Sometimes
10	10	M	49	1	2–3	155	Sometimes
11	15	F	50	2–3	Everyday	510	Never
12^a^	15	F	45	1	1	450	Often
13^a^	8	M	22	1	1	235	Sometimes
14^a^	11	F	25	1–2	1–2	251	Often
15	15	M	54	1–2	Everyday	192	Never
16^a^	8	M	34	2	5–6	260	Sometimes
17	8	M	26	2	Everyday	190	Sometimes
18^a^	12	M	32	2	5	350	Sometimes
19^a^	11	F	34	1	Everyday	165	Sometimes
20^a^	8	M	40	1	1	90	Often
21^a^	13	M	41	1	2–3	245	Often
22^a^	10	F	31	1	3–4	310	Sometimes
23^a^	7	F	23	1–2	6	115	Sometimes
24^a^	11	M	37	1	6	120	Sometimes
25^a^	10	M	41	1–2	1–2	153	Often
26^a^	10	M	50	1	3–4	304	Never

All patients wet the bed only at night. The age is given in years; M = male; F = female.

a These children can wake up after bed-wetting.

b The bladder volume was defined by b-ultrasound results: children with PMNE were asked to try their best to hold back urine for three times while their bladder volumes were measured by b-ultrasound. The largest measured value was defined as the bladder volume.

### 4.3 Data acquisition

MRI data were acquired on a Siemens 3 T Trio MR scanner with a 12-channel phased array coil. The DTI acquisition used a single-shot spin-echo planar imaging sequence in contiguous axial planes covering the whole brain. The diffusion sensitizing gradients were applied along 12 non-collinear directions together with an acquisition without diffusion weighting (b = 0). Imaging parameters were set to the following values: TR = 6600 ms, TE = 89 ms, average = 4, b-value = 1000 s/mm^2^, slice thickness = 2.5 mm, and 50 slices. Matrix size was acquired with a 128×128 and reconstructed to 256×256. The resolution was 2×2×2.5 mm^3^. The subjects were told not to move during the scans, and the acquisition time was 6 minutes and 5 seconds.

### 4.4 Data processing and statistical analysis

#### Preprocessing

SPM 8 (http://www.fil.ion.ucl.ac.uk/spm/), Matlab 2009 (The MathWorks, Natick, MA) and FSL4.1 (http://www.fmrib.ox.ac.uk/fsl/) were used to analyze the data. First, the DICOM files of each DWI acquisition were converted into a single multivolume NIFTI file. Then, FSL's “eddy current correction” was used to correct the distortions induced by the eddy current and head motion in the dataset. The brain was extracted using BET (Brain Extraction Tool, http://fsl.fmrib.ox.ac.uk/fsl/bet2/) for further processing steps. Finally, the FA and MD maps were calculated with FSL DTIFit (http://www.fmrib.ox.ac.uk/fsl/fdt/fdt_dtifit.html).

#### SPM analysis

A whole brain voxel-wise analysis was performed using SPM 8. First, to reduce the potential errors caused by the adult template, we used the FA maps from our control group to generate our FA-specific pediatric template, which included following steps:

1. We normalized the FA maps of the control group based on the deformation information that was generated from the corresponding unweighted images (first b = 0 image) and the echo-planar imaging (EPI) template (in MNI152 space) in SPM; we called these the wFA maps.
2. These wFA maps were averaged to generate a mean map.
3. The mean map was smoothed (using a [6,6,6] FWHM Gaussian kernel) to obtain our FA-specific pediatric template for further analysis [43]. Subsequently, we normalized all of the original FA maps (from both patient and control groups) based on the deformation field produced from both the original FA maps and our FA-specific pediatric template. Then, the normalized FA maps were smoothed (using a [6,6,6] filter) for the statistical analysis. Finally, a fractional design specification was set up to compare the PMNE group against the controls using a two-sample t-test. The same processes were applied to the MD maps. For all analyses, the statistical maps were thresholded at p<0.005 (uncorrected). Moreover, an extent threshold of 10 contiguous voxels was applied to exclude small clusters that emerged by chance [44].

#### ROI analysis

To confirm the significant differences in the thalamus seen in PMNE children and unaffected controls, we also performed a ROI analysis. Each child's thalamus mask was manually drawn on one of the FA slices according to the cerebral surgery expert's opinion; the thalamus was visualized to be the biggest and the most distinct (Fig. 2C). Because the FA and the related MD maps are generated from the same individual DTI dataset, the ROIs were translated onto the corresponding MD maps. Finally, the mean values of FA and MD in all ROIs were calculated using Matlab, and a two-sample t-test was carried out between the two groups.
